# Improved Working Memory but No Effect on Striatal Vesicular Monoamine Transporter Type 2 after Omega-3 Polyunsaturated Fatty Acid Supplementation

**DOI:** 10.1371/journal.pone.0046832

**Published:** 2012-10-03

**Authors:** Rajesh Narendran, William G. Frankle, Neale S. Mason, Matthew F. Muldoon, Bita Moghaddam

**Affiliations:** 1 Department of Radiology, University of Pittsburgh, Pittsburgh, Pennsylvania, United States of America; 2 Department of Psychiatry, University of Pittsburgh, Pittsburgh, Pennsylvania, United States of America; 3 Department of Medicine, University of Pittsburgh, Pittsburgh, Pennsylvania, United States of America; 4 Department of Neuroscience, University of Pittsburgh, Pittsburgh, Pennsylvania, United States of America; Centre for Addiction and Mental Health, Canada

## Abstract

Studies in rodents indicate that diets deficient in omega-3 polyunsaturated fatty acids (n–3 PUFA) lower dopamine neurotransmission as measured by striatal vesicular monoamine transporter type 2 (VMAT2) density and amphetamine-induced dopamine release. This suggests that dietary supplementation with fish oil might increase VMAT2 availability, enhance dopamine storage and release, and improve dopamine-dependent cognitive functions such as working memory. To investigate this mechanism in humans, positron emission tomography (PET) was used to measure VMAT2 availability pre- and post-supplementation of n–3 PUFA in healthy individuals. Healthy young adult subjects were scanned with PET using [^11^C]-(+)-α-dihydrotetrabenzine (DTBZ) before and after six months of n–3 PUFA supplementation (Lovaza, 2 g/day containing docosahexaenonic acid, DHA 750 mg/d and eicosapentaenoic acid, EPA 930 mg/d). In addition, subjects underwent a working memory task (n-back) and red blood cell membrane (RBC) fatty acid composition analysis pre- and post-supplementation. RBC analysis showed a significant increase in both DHA and EPA post-supplementation. In contrast, no significant change in [^11^C]DTBZ binding potential (BP_ND_) in striatum and its subdivisions were observed after supplementation with n–3 PUFA. No correlation was evident between n–3 PUFA induced change in RBC DHA or EPA levels and change in [^11^C]DTBZ BP_ND_ in striatal subdivisions. However, pre-supplementation RBC DHA levels was predictive of baseline performance (i.e., adjusted hit rate, AHR on 3-back) on the n-back task (y = 0.19+0.07, r^2^ = 0.55, p = 0.009). In addition, subjects AHR performance improved on 3-back post-supplementation (pre 0.65±0.27, post 0.80±0.15, p = 0.04). The correlation between n-back performance, and DHA levels are consistent with reports in which higher DHA levels is related to improved cognitive performance. However, the lack of change in [^11^C]DBTZ BP_ND_ indicates that striatal VMAT2 regulation is not the mechanism of action by which n–3 PUFA improves cognitive performance.

## Introduction

Previous studies in humans suggest that n–3 PUFA deficiency is associated with impairment in mood [1] and cognitive functioning [2]. Some [3]–[5], but not all studies [6]–[9] suggest that the supplementation of n–3 PUFA in several neuropsychiatric disorders such as mood disorders, schizophrenia and attention deficit hyperactivity disorder holds promise as a primary or adjunctive therapy. Mechanistic studies are discovering roles of n–3 PUFAs in modulation of neuronal membrane fluidity and permeability, enhancement of monoamine transmission, alteration of the activity of protein kinases and phosphatidylinositol-associated second messenger systems, alteration in gene expression and decreased oxidative stress and inflammation. Nonetheless, how these actions relate to the putative effects of n–3 PUFA on cognitive functioning and affective symptoms is unknown.

Basic science investigations involving rodents indicate that n–3 PUFA deficiency alters the transmission of monoamines such as dopamine and serotonin in the brain [10]. For example, studies that have measured stimulant-induced dopamine release report 35% and 60–80% reductions in dopamine release in the ventral striatum and prefrontal cortex respectively in n–3 PUFA deficient animals relative to controls [11], [12]. Also compelling are the tyramine-induced dopamine release microdialysis studies that have reported a 90% reduction in prefrontal cortical dopamine transmission [13], [14] and the cerebral monoamine quantitation studies that have reported a 40 to 75% reduction in prefrontal dopamine in n–3 PUFA deficient animals relative to controls [15], [16]. In addition, rodent studies are consistent in reporting a 25 to 60% reduction in the VMAT2 density in the prefrontal cortex and ventral striatum in n–3 PUFA deficient animals relative to controls [11], [12], [14], [17]. Since most of these studies involved pregnant rodents and pups the effects of n–3 PUFA supplementation on dopamine in a mature animal/healthy human are not known. Nevertheless, as VMAT2 regulates the size of the vesicular dopamine pool available for release into the synapse, it is plausible that n–3 PUFA increases dopamine transmission by increasing the number of dopamine storage vesicles and associated VMAT2. Therefore it is tempting to speculate that dietary supplementation with fish oil enriched in n–3 PUFA increases VMAT2 availability, in turn enhancing dopamine storage and release and improving dopamine-dependent cognitive and mood functions in a broad array of neuropsychiatric disorders.

To evaluate this hypothesis we evaluated 11 healthy individuals with the selective VMAT2 PET radioligand, [^11^C]DTBZ both before and after six-months of n–3 PUFA supplementation (Omega-3-acid ethyl esters, Lovaza 2 g/day, which contains DHA 750 mg/d and EPA 930 mg/d). Our primary hypothesis was that n–3 PUFA would increase VMAT2 availability (measured as [^11^C]DTBZ binding potential, BP_ND_) in healthy individuals after six months of supplementation. In addition, we hypothesized that this increased availability of VMAT2 will lead to greater vesicular dopamine stores and improve dopamine-dependent working memory, which was measured using a verbal n-back task and three working memory loads (1-back, 2-back and 3-back).

## Materials and Methods

### Ethics Statement

The study was conducted following the approvals of the University of Pittsburgh Institutional Review Board and Radioactive Drug Research Committee. All subjects provided written informed consent.

Study criteria for healthy controls were [1] males or females between 18 and 25 years old, of all ethnic and racial origins; [2] no past or current Diagnostic and Statistical Manual of Mental Disorders IV criteria for psychiatric disorders, including addiction to drugs, alcohol or nicotine (as confirmed by urine drug screen at screening) [3] not currently on any prescription or over the counter medications including vitamins or herbal supplements; [4] female subjects were not currently pregnant and used of an effective birth control such as intrauterine contraceptive device, oral contraceptive pills during the entire course of the study; [5] no current or past severe medical or neurological illnesses (including glaucoma, seizure disorders, a focal finding on magnetic resonance imaging, MRI such as stroke or tumor) as assessed by a complete medical assessment; [6] no hypersensitivity to fish or shell fish; [7] no history of significant radioactivity exposure (nuclear medicine studies or occupational exposure); [8] no metallic objects in the body that are contraindicated for MRI; [9] no drinking of more than two standard alcoholic drinks per day; [10] no first degree relatives with an Axis I psychiatric disorder; [11] no consumption of fish more than twice a month or currently on fish oil supplements.

A total of thirteen subjects who met inclusion/exclusion criteria (as determined by a structured clinical interview for DSM IV, medical evaluation, electrocardiogram, and routine blood and urine tests, which included a drug screen and pregnancy test) were enrolled to participate in the study that was conducted in the outpatient setting. All enrolled subjects underwent a pre-supplementation [^11^C]DTBZ PET scan, n-back task and RBC membrane extraction for fatty acid analysis after an overnight fast. Then, subjects were supplemented with n–3 PUFA (Lovaza), 2 g once daily for six months after which they were eligible for the post-supplementation evaluations (conducted a minimum of five months and maximum of 6 months after n–3 PUFA supplementation), which included a repeat [^11^C]DTBZ PET scan, n-back task and RBC for fatty acid analysis. Subjects were monitored on a monthly basis during the n–3 PUFA supplementation period for subjective complaints, medication adherence (via pill counts), illicit drug abuse and pregnancy. In addition to this subjects underwent a complete blood cell count, liver function test, fasting lipid profile and RBC for fatty acid analysis at 1-month, 3-months, 5-months to monitor for abnormal lab results (such as elevations in liver function tests and low-density lipoproteins and reduction in platelet count) and confirmed adherence to n–3 PUFA. The monitoring led to discontinuation on n–3 PUFA supplementation in two individuals –one for elevated low-density lipoproteins (193 mg/dl) and another for low platelet counts (120,000/µL) at 1-month and 5-months post-supplementation respectively (these abnormal laboratory values reverted back to normal range after discontinuation in both these subjects). Thus, the final sample includes pre- and post-supplementation data in only 11 out of the 13 enrolled subjects.

### RBC Fatty Acid Composition

Fasting blood samples were processed for the separation of RBC membranes using previously methods and stored at −80 degree Celsius [18]. These frozen RBC samples were analyzed for fatty acid composition using gas chromatography [19]. Individual PUFA levels are expressed as percentages of the total fatty acid pool (weight or mol %).

### Working Memory Assessment

We chose to assess verbal working memory based on a previous study that showed a relationship between this neurocognitive domain and serum DHA [2]. The choice was also driven by the literature that supports a role for dopamine in working memory [20]–[23]. Working memory assessment was performed using a verbal n-back task that used three loads of working memory (1-back, 2-back and 3-back) consistent with that previously reported in [20]. The outcome measure for the n-back was the adjusted hit rate (AHR, range −1 to 1), which was calculated as the difference between hit rate (correct responses/number of targets, range 0 to 1) and error rate (errors/number of non targets, range 0 to 1).

### [^11^C]DTBZ PET Imaging

Prior to PET imaging, a magnetization prepared rapid gradient echo structural MRI scan was obtained using a Siemens 3 Tesla Trio scanner for determination of regions of interest.

[^11^C]DTBZ was synthesized using the methodology reported previously by Kilbourn, et al. [24]. PET imaging sessions were conducted with the ECAT EXACT HR+ camera. [^11^C]DTBZ was injected as a bolus plus constant infusion consistent with that reported in [25], [26] because this infusion paradigm allows for radioactivity to be measured at true equilibrium, thereby eliminating the need for invasive arterial catheterization (i.e., the amount of radiotracer in the region of interest, reference region, arterial and venous compartments are at equilibrium). Briefly, 55% of the [^11^C]DTBZ dose (∼ 15 mCi) was administered as an intravenous bolus injection over the first 30 seconds, while the remaining 45% of the dose was continuously infused over the next 60 minutes. This infusion ratio allowed for [^11^C]DTBZ to reach steady state thirty minutes after the beginning of the bolus plus constant infusion [25], [26]. Following a 10 minute transmission scan, emission data were then collected in 3D mode from time, t = 30 to 60 minutes, relative to the start of the bolus plus constant infusion in a series of six consecutive 5-minute frames to correspond to steady state concentration for [^11^C]DTBZ. In addition, four venous blood samples (collected at time = 30, 40, 50 and 60 min) were obtained to measure plasma concentration of [^11^C]DTBZ as described in [27]. Parent compound plasma concentrations in these four samples were averaged to obtain [^11^C]DTBZ concentration at steady state (C_SS,_ µCi/mL). Plasma clearance (C_L_, L/h) was calculated as the rate of infusion/C_SS_ and the free fraction (fp) was measured using previously described methods [26], [28], [29].

All region drawing and image analysis was performed with MEDx (Sensor Systems, Inc., Sterling, Virginia) and SPM2. Regions of interest were drawn on the MRI and transferred to the co-registered PET scan. The primary region of interest, the striatum was divided into five anatomical and three functional subdivisions using criteria outlined in [30]. The three functional subdivisions of the striatum included the limbic striatum (ventral striatum), the associative striatum (which, included the precommissural caudate, precommissural putamen and postcommissural caudate) and sensori-motor striatum (postcommissural putamen). The occipital cortex was used as the reference region [26], [28]. Correction for head movement and co-registration of the PET data to the MR were done using methods described in [31].

In this section we use the consensus nomenclature for in vivo imaging of reversibly binding radioligands to describe all outcome measures [32]. The regional tissue distribution volume (V_T ROI_, mL/cm^3^) defined as the ratio of [^11^C]DTBZ concentration in the region of interest (C_T_, µCi/cm^3^) to the concentration of un-metabolized [^11^C]DTBZ in venous plasma (C_SS_, µCi/g) at equilibrium was derived as.
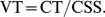



The concentration of VMAT2 is negligible in the occipital cortex [26], [28], such that only free and nonspecifically bound radiotracer is considered to contribute to V_T_ in the occipital cortex (V_T OCC_ ). Thus, V_T__OCC_ was assumed to be equal to the non-displaceable distribution volume (V_ND_).

VMAT2 availability in the striatal regions of interest was estimated as [^11^C]DTBZ BP_ND_, i.e., binding potential relative to non-displaceable uptake. This was computed as.
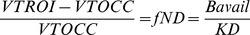
where f_ND_ is the free fraction of radiotracer in brain expressed relative to the non-displaceable concentration (f_ND_  =  f_p/_V_ND_), B_avail_ is the density of VMAT2 available to bind to [^11^C]DTBZ in vivo and K_D_ is the equilibrium disassociation constant of [^11^C]DTBZ.

The effect of n–3 PUFA supplementation on VMAT2 availability was calculated as the relative change in BP_ND_ (%).
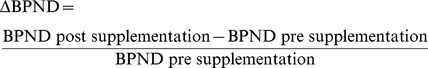



### Statistical Analysis

All statistical analyses were performed using IBM SPSS statistics, version 20. Comparison of the pre- and post- supplementation condition outcome measures such as RBC PUFA, AHR, Δ BP_ND_ etc., were evaluated with paired t tests and linear mixed model with region of interest as a repeated measure and condition as fixed factor. Relationships between the fatty acid composition, cognitive and imaging measures were analyzed with Pearson product moment correlation coefficient. A two-tailed probability value of p<0.05 was selected as significant.

## Results

11 subjects (5 males/6 females; all Caucasian) completed the study. The mean age of the subjects was 22±2 years. The mean body mass index of the subjects was 25.6±3.5. All eleven subjects were non-smokers.

### RBC Fatty Acid Composition

**Figure 1:**
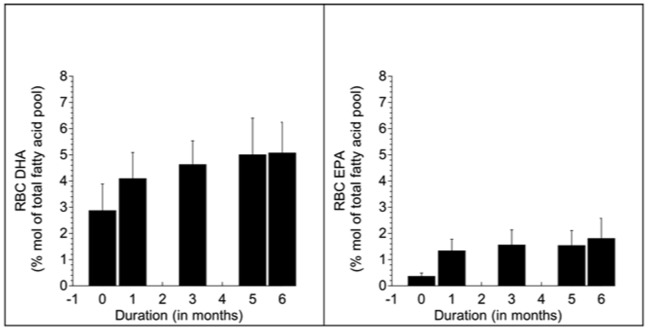
A and B show the increase in RBC DHA and EPA over the course of the six-month study, i.e., from pre-supplementation levels at baseline (0-month) to post-supplementation levels prior to the [11C]DTBZ PET scan (6-months).

The results of the RBC fatty acid composition analysis before and after six months of n–3 PUFA supplementation are shown in Table 1. They include the main n–3 PUFAs (DHA, EPA) and its precursor α-linolenic acid (ALA) and the main n–6 PUFA (arachidonic acid, AA) and its precursor linolenic acid (LA). Compared to the pre-supplementation condition, n–3 PUFA led to mean increases in RBC DHA and EPA of 75% and 450% respectively, and decreases in AA of 13% at six months (p<0.05, paired t tests, Table 1). No significant changes were observed in the n–3 and n–6 PUFA precursors ALA and LA. Figure 1 A and B show the increase in RBC DHA and EPA over the 6-month duration of the study.

**Table 1:** RBC fatty acid composition analysis.

Type	PUFA	Pre- n3PUFA	Post- n3PUFA	t	df	p-value
n–3 PUFA	ALA	0.4±0.1	0.4±0.1	0.11	10	0.92
	DHA	2.9±1.0	5.1±1.2	−9.89	10	<0.01
	EPA	0.4±0.1	1.8±0.8	−6.30	10	<0.01
n–6 PUFA	LA	21.7±5.1	21.9±3.8	−0.13	10	0.90
	AA	13.9±2.0	12.2±2.0	3.49	10	0.01

Values are mean and standard deviation (SD), n = 11 per condition.

p-values are from two-tailed, paired t tests; t is t statistic; df is degrees of freedom.

### Working Memory Assessment

Table 2 shows the AHR for 1-, 2- and 3-back conditions before and after n–3 PUFA supplementation. n–3 PUFA supplementation improved working memory performance (measured as AHR) in the 3-back (p<0.05, paired t test, Table 2), but not in the 1- and 2- back conditions.

**Table 2:** Adjusted hit rate from the n-back working memory task.

n-back	Pre- n3PUFA	Post- n3PUFA	t	df	p-value
1-back	0.98±0.04	0.99±0.02	−1.480	10	0.17
2-back	0.93±0.10	0.94±0.09	−0.399	10	0.70
3-back	0.65±0.27	0.80±0.15	−2.292	10	0.04

Values are mean and standard deviation (SD), n = 11 per condition.

p-values are from two-tailed, paired t tests; t is t statistic; df is degrees of freedom.

**Figure 2:**
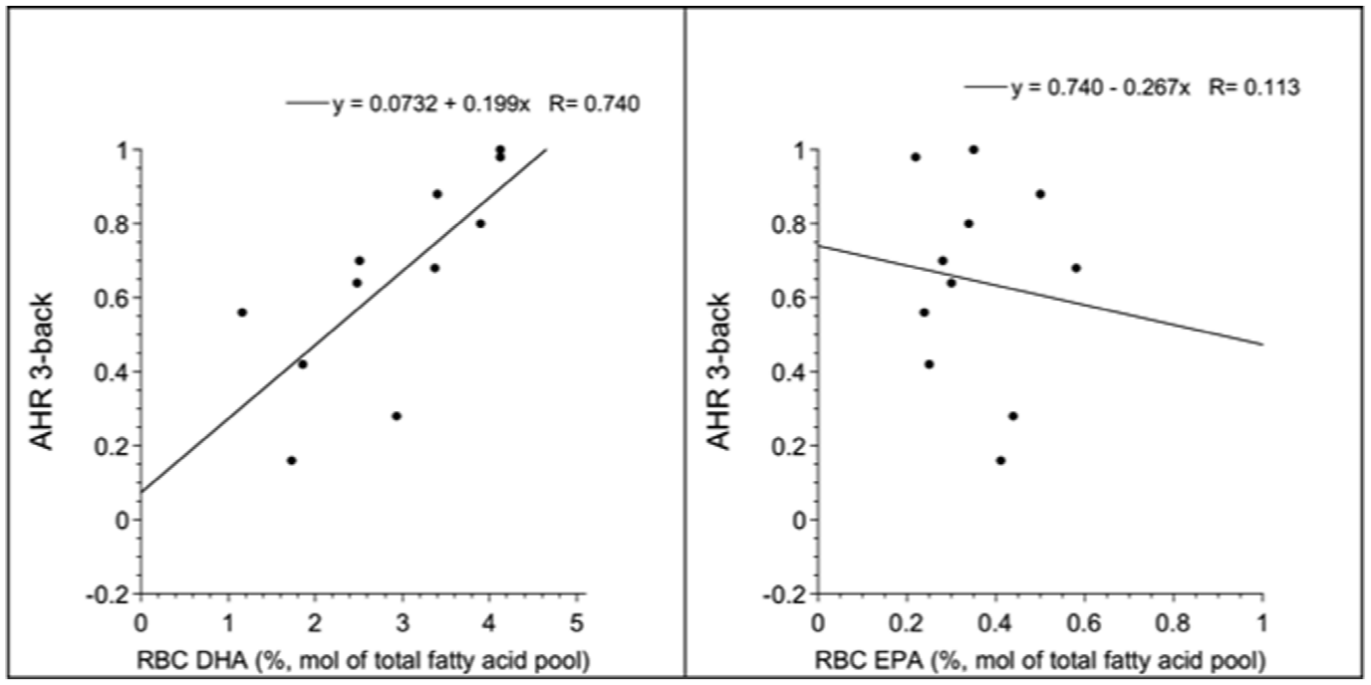
Shows the relationship between pre-supplementation RBC DHA or EPA in x-axis and pre-supplementation performance (AHR) in 3-back test in y-axis. The AHR ranges from 1 (best performance) to −1 (worst performance), with a score of 0 corresponding to performance at chance level. RBC DHA (Panel A), but not EPA (Panel B) was associated with performance in the task.

The pre-supplementation AHR on the 3-back was linearly related to pre-supplementation RBC DHA (r = 0.74, p = 0.009, see Figure 2A), but not EPA (r = −0.11, p = 0.76, see Figure 2B).

The post-supplementation AHR on the 3-back was not related to the post-supplementation RBC DHA (r = −0.06, p = 0.86) or EPA levels (r = −0.13, p = 0.71).

There was no significant association between the change in working memory performance (Δ AHR on 3-back) and change in RBC DHA (r = 0.29, p = 0.39) or EPA (r = 0.04, p = 0.90) levels following supplementation.

### [^11^C]DTBZ PET Imaging

Critical PET scan parameters are listed in Table 3.

**Table 3:** Scan parameters.

Parameter	Pre- n3PUFA	Post- n3 PUFA	t	df	p-value
Injected dose (mCi)	16.0±0.6	16.0±0.5	−0.09	10	0.93
SA (Ci/mmoles)	2973±1543	3189±1288	−0.35	10	0.73
Injected Mass (ug)	2.8±2.6	2.1±1.0	0.86	10	0.41
Free Fraction,fp (%)†	30.2±2.9	30.9±2.8	−0.57	9	0.58
Clearance(L/h)†	72.3±17.4	67.4±21.0	0.70	9	0.50
Occipital VT(mL cm-3)†	3.80±0.89	3.38±1.05	1.31	9	0.22

Values are mean and standard deviation (SD), n = 11 per condition (unless noted as different).

p-values are from two-tailed, paired t tests; t is t statistic; df is degrees of freedom.

† n = 10/condition.

[^11^C]DTBZ injected dose, specific activity at time of injection, and injected mass did not differ between the pre- and post-n–3 PUFA supplementation conditions. No significant between-condition differences were observed in the plasma free fraction and clearance rate of [^11^C]DTBZ, or in [^11^C]DTBZ occipital cortex distribution volume, V_ND_ measure (data available from n = 10/11 subjects, in whom venous line placement was successful).

n–3 PUFA supplementation had no significant effect on [^11^C]DTBZ BP_ND_ in the striatal subdivisions [linear mixed model, effect of condition, F(1,20) = 0.52, p = 0.48; effect of region, F(4, 80) = 285.6: p<0.001; condition-by-region interaction, F(4, 80) = 0.63, p = 0.64]. In addition, a test of between-condition differences in each region of interest failed to reach significance in all five striatal subdivisions (p>0.05, paired t tests, data in Table 4).

**Table 4:** Regional [**^11^**C]DTBZ binding potential (BP_ND_).

Functional subdivison	Anatomical subdivisoon	Pre- n3 PUFA	Post- n3 PUFA	DELTA BP_ND_	t	df	p-value
Limbic striatum	Ventral Striatum	1.69±0.11	1.62±0.18	−4.1±8.8	1.53	10	0.16
Associative striatum		1.82±0.18	1.78±0.22	−1.9±7.6	0.79	10	0.45
	Precommisural dorsal caudate	1.83±0.19	1.80±0.23	−1.8±7.6	0.78	10	0.46
	Postcommissural caudate	1.44±0.19	1.43±0.27	−1.0±10.1	0.24	10	0.82
	Precommisural anterior putamen	2.09±0.21	2.03±0.22	−2.8±7.3	1.30	10	0.22
Sensori-motor striatum	Postcommisural putamen	2.50±0.23	2.39±0.22	−3.9±8.2	1.54	10	0.15
Whole striatum		2.03±0.18	1.96±0.21	−3.1±7.7	1.26	10	0.24

Values are mean and standard deviation (SD), n = 11 per condition.

Associative striatum values are a weighted average of Precommissural dorsal caudate, Postcommissural caudate, and Precommissural anterior putamen; Whole striatum values are a weighted average of the five anatomical subdivisions.

p-values are from two-tailed, paired t tests; t is t statistic; df is degrees of freedom.

Correlation analyses revealed no significant relationship between pre-supplementation [^11^C]DTBZ BP_ND_ in the striatum and RBC DHA (r = −0.40, p = 0.22) or EPA (r = 0.12, p = 0.70) levels. Also, no significant associations were noted between the change in [^11^C]DTBZ BP_ND_ in the striatum and change in RBC DHA (r = −0.29, p = 0.39) or EPA (r = −0.04, p = 0.90) levels following supplementation. No significant associations were noted when the above correlations were performed using [^11^C]DTBZ BP_ND_ and Δ BP_ND_ from the functional or anatomical subdivisions of the striatum.

## Discussion

In this study, we evaluated VMAT2 availability with [^11^C]DTBZ and PET in a group of healthy young adults before and after six months of supplementation of a FDA approved formulation of n–3 PUFA (Lovaza, 2 g/day). Despite the fact that the formulation used in this study led to significant elevations in RBC DHA (1.75-fold) and EPA (4.5-fold) levels relative to pre-supplementation values, we failed to detect an effect for it on striatal VMAT2 availability. The mean change in [^11^C]DTBZ BP_ND_ in the striatal subdivisions (range −1 to −4%) after n–3 PUFA supplementation was well within the reported test-retest variability (4 to 7%) for this radioligand [28]. This observation in humans is somewhat inconsistent with rodent studies that suggest n–3 PUFA deficient animals relative to controls have 25 to 60% less VMAT2 binding in the ventral striatum [12]–[14]. An important difference that led to the inability to detect an effect on [^11^C]DTBZ binding might be related to the fact that healthy humans were supplemented with n–3 PUFA in this study, as opposed to the rodent studies in which a group of animals were developmentally deprived of n–3 PUFA and compared to controls**.** Thus, the possibility of dietary depletion of n–3 PUFA leading to a reduction in striatal VMAT2 availability in humans cannot be excluded based on the six-month supplementation data. Because individuals with diets deficient in n–3 PUFA are likely to have less RBC DHA/EPA, we evaluated whether lower RBC DHA/EPA levels are associated with lower striatal VMAT2 availability in subjects before supplementation. Contrary to this hypothesis, we found no relationship between the RBC DHA/EPA levels and striatal [^11^C]DTBZ BP_ND_. Taken together these data do not support an effect for n–3 PUFA on striatal VMAT2 in healthy adults.

Two interesting observations are reported in this study. The first is that in this group of young adults superior working memory performance in the 3-back condition prior to supplementation was correlated with higher RBC DHA. This finding is consistent with a previous report in which higher serum DHA was related to superior performance on tests of non verbal reasoning and working memory in a relatively large cohort of middle aged adults [2]. Second, there was an improvement in working memory performance in the 3-back condition after six months of n–3 PUFA supplementation. Although, practice-effects cannot be ruled out as the reason for this observation in this cohort, this result is consistent with some clinical trials suggesting that n–3 PUFA (fish oil) supplementation improves cognitive functioning in elderly adults with mild to no cognitive impairment [33]–[37]. Surprisingly, 3-back performance improvement was significant despite the fact that there was no correlation between changes in AHR and RBC DHA/EPA levels following supplementation with n–3 PUFA. But, when individuals were stratified into two groups based on their pre-supplementation DHA levels (i.e., less than or greater than 3% mol of total fatty acid pool) we found that the mean change in AHR 3-back was 0.29±0.18 in the low DHA group (n = 6 subjects) and −0.01±0.14 in the high DHA group (n = 5 subjects). This suggests that the individuals with low pre-supplementation DHA levels benefitted the most by the n–3 PUFA. Further investigation in larger samples is needed to understand this relationship.

The fact that working memory performance was enhanced by n–3 PUFA supplementation regardless of an effect on striatal VMAT2 suggests that its potential pro-cognitive effects, are mediated via extrastriatal dopamine or other non-dopaminergic mechanisms such as effects on inflammation, cellular signaling and trafficking etc. Alternatively other mechanisms that govern the release and storage of dopamine such as afferent regulation of dopamine cell activity or dopamine synthesis may play a role. Future studies are needed to investigate the role of n–3 PUFA on dopamine release mechanisms as well as indices of prefrontal cortical dopamine function. The latter studies are especially critical because prefrontal cortical dopamine is linked to working memory performance [38]. Since the concentration of dopamine in the prefrontal cortex is 10 to 35-fold lower than in the striatum (dopamine concentration: cortex 0.5 nM vs striatum 5–18 nM) it is likely that a relatively small increase in dopamine following n–3 PUFA supplementation has a greater impact in the cortex and translates to pro-cognitive effects [39], [40]. In addition, the likelihood to detect relatively small changes in dopamine concentration is better in the prefrontal cortex than in the striatum because of the low baseline dopamine levels in this region. Future studies with D_1_ and D_2/3_ receptor PET radiotracers to evaluate the effects of n–3 PUFA on prefrontal cortical dopamine and its relationship with working memory are necessary to address these issues.

The current investigation was designed as a proof of concept study to clarify whether n–3 PUFA supplementation leads to increased VMAT2 availability in the human striatum. This question arose based on a recent PET imaging study in which we showed that cocaine addicts have lower vesicular monoamine transporter type 2 in the striatum relative to healthy controls [41]. This reduction in VMAT2, which suggests fewer dopamine storage vesicles in the terminals, is one of the mechanisms that lead to the blunted (or less) dopamine release in the striatum after a psychostimulant challenge in cocaine addicts compared to controls [42]. In addition, more recent data links this blunted dopamine release in the striatum to relapse and treatment failure in cocaine addicts [43], [44]. Since preclinical studies in rodents signaled that diets deficient in n–3 PUFAs lead to lower striatal VMAT2 density by 25 to 60% and reduce stimulant-induced DA release [10] we were interested in evaluating the potential of n–3 PUFA as means to increase VMAT2 availability, enhance DA storage and release, and prevent relapse in cocaine addicts. The result of this human imaging study suggests that n–3 PUFA supplementation is unlikely to enhance striatal DA transmission in cocaine addicts and promote abstinence.

In summary, we found no effect for n–3 PUFA supplementation on striatal VMAT2 availability in healthy humans using [^11^C]DTBZ and PET. Higher RBC DHA levels were associated with better working memory performance in this cohort of young adults, which is consistent with that previously shown in middle-aged adults. Also, n–3 PUFA supplementation improved working memory performance, which is consistent with some but not all clinical trials that have evaluated the pro-cognitive effects of n–3 PUFA in humans. Further research is warranted to elucidate the mechanisms by which n–3 PUFA enhances cognitive performance in healthy individuals.
